# Pseudoaneurisma da artéria temporal superficial: relato de três casos

**DOI:** 10.1590/1677-5449.009517

**Published:** 2018

**Authors:** Marcio Miyamotto, Matheus Schimidt Evangelista, Victor Hugo Granella, Bruna Zimmerman Angelo, Bárbara Milena Marciniak, Pedro Henrique Hennig, Ian Gimenez Ribeiro, Ricardo César Rocha Moreira

**Affiliations:** 1 Pontifícia Universidade Católica do Paraná –PUC-PR, Hospital Universitário Cajuru – HUC, Serviço de Cirurgia Vascular e Endovascular, Curitiba, PR, Brasil.; 2 Instituto VESSEL de Aperfeiçoamento Endovascular de Curitiba, Curitiba, PR, Brasil.; 3 Hospital Nossa Senhora das Graças – HNSG, Serviço de Cirurgia Vascular e Endovascular Elias Abrão, Curitiba, PR, Brasil.; 4 Pontifícia Universidade Católica do Paraná – PUC-PR, Hospital Universitário Cajuru – HUC, Liga Acadêmica de Medicina Vascular – LAMEV, Curitiba, PR, Brasil.

**Keywords:** artérias temporais, falso aneurisma, traumatismos craniocerebrais

## Abstract

O pseudoaneurisma da artéria temporal superficial é um evento raro. Ocorre principalmente em homens jovens em decorrência de traumatismo craniano fechado. A maioria dos casos é assintomática, podendo eventualmente haver associação com sintomas vagos. A rotura do pseudoaneurisma e o desenvolvimento de déficits neurológicos são complicações esporádicas. Os autores relatam três casos de pseudoaneurisma de artéria temporal superficial pós-traumatismo craniano fechado. Os pacientes foram manejados com sucesso por ligadura e ressecção dos pseudoaneurismas.

## INTRODUÇÃO

 O pseudoaneurisma traumático da artéria temporal superficial (ATS) é raro e representa menos de 1% dos aneurismas descritos 1 . O primeiro caso foi relatado por Thomas Bartholin em 1740 2 ^,^ 3 e, desde então, aproximadamente 200 casos foram descritos na literatura 4 . Embora associado à baixa morbidade, o pseudoaneurisma da ATS pode causar uma série de sintomas locais e até mesmo rotura. Os autores relatam três casos de pseudoaneurisma da ATS secundários a traumatismo craniano fechado. 

## DESCRIÇÃO DOS CASOS

### Caso 1

**Figura 1:**
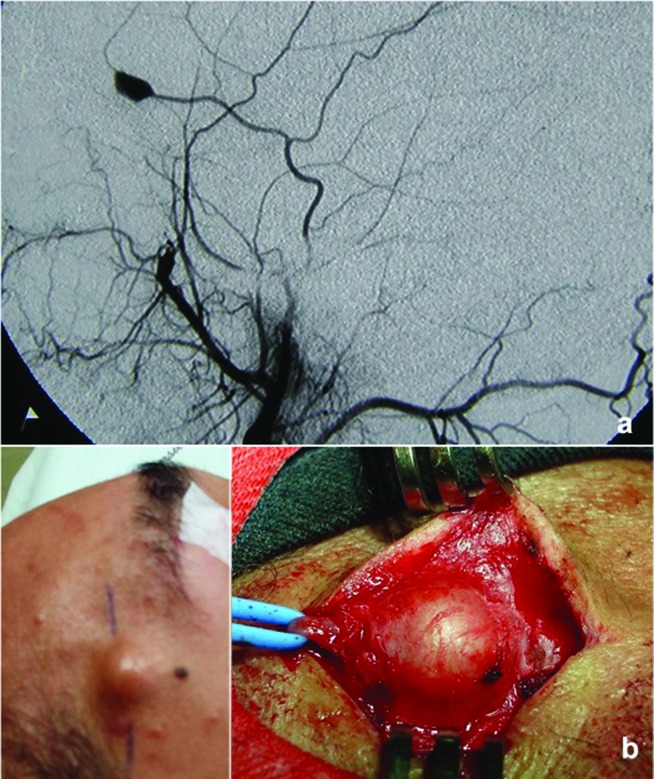
Caso 1. (a) Arteriografia evidenciando pseudoaneurisma do ramo anterior da artéria temporal superficial; (b) Tratamento cirúrgico através de ligadura arterial e ressecção do pseudoaneurisma.

 Paciente do sexo masculino, 22 anos, com queixa de desconforto na região frontoparietal direita associado a um aumento de volume local. Relatava história de acidente automobilístico havia três meses com trauma contuso na região temporal direita. Referiu sangramento no momento do trauma e desenvolvimento de hematoma periorbitário. Foi avaliado e manejado com sutura da ferida e observação; porém, após 20 dias, notou um aumento progressivo de volume na região do trauma. Ao exame físico, o paciente apresentava nódulo pulsátil de aproximadamente 1,5 cm de diâmetro, sem sopro ou frêmito, com diminuição da pulsatilidade à manobra de compressão proximal. Com o diagnóstico de pseudoaneurisma da ATS, foi realizada uma arteriografia para afastar lesões associadas, que confirmou o diagnóstico e eliminou a presença de lesões intracranianas ( Figura 1 a). O paciente foi submetido a tratamento cirúrgico por ligadura e ressecção do aneurisma ( Figura 1 b). A evolução foi favorável e não houve recorrência do aneurisma. 

### Caso 2

**Figura 2:**
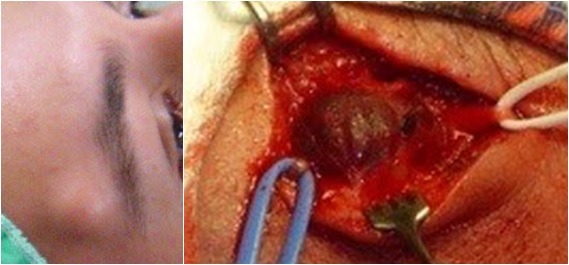
Tratamento cirúrgico do segundo caso por ligadura arterial e aneurismectomia.

 Paciente do sexo masculino de 27 anos, com história de agressão direta (soco) em região frontoparietal 15 dias antes. Desenvolveu uma massa pulsátil indolor na região temporal diagnosticada como pseudoaneurisma de ATS, manejada com ressecção cirúrgica e ligadura arterial. O paciente apresentou boa evolução, sem recorrência do aneurisma ( Figura 2 ). 

### Caso 3

**Figura 3:**
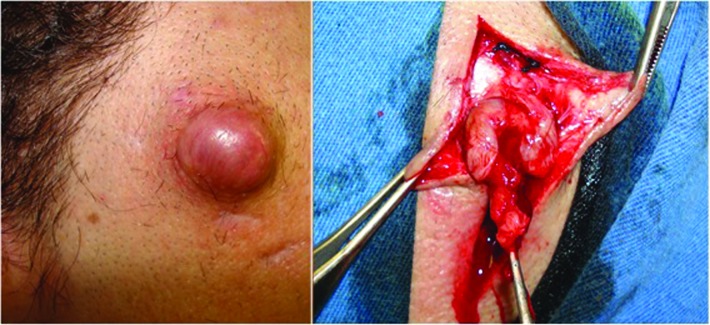
Tratamento cirúrgico do terceiro caso por ligadura arterial e ressecção do pseudoaneurisma.

 Paciente do sexo masculino de 29 anos, vítima de acidente automobilístico de alta energia. Ainda em cuidados intensivos devido ao coma, foi evidenciado aumento de volume na região temporal direita diagnosticado como pseudoaneurisma. Foi submetido ao tratamento cirúrgico com boa evolução ( Figura 3 ). 

## DISCUSSÃO

 Após a primeira descrição, várias séries de pseudoaneurismas da ATS têm sido relatadas na literatura. Embora tenha sido primariamente descrito após trauma penetrante 5 , a grande maioria dos casos de pseudoaneurisma da ATS ocorre em decorrência de traumatismo fechado 6 . Está associado à prática de vários esportes como hóquei, rúgbi, *squash* e beisebol. Também é descrito associado a lesões penetrantes por artroplastia temporomandibular, transplante de cabelo, remoção de cistos, lacerações e ferida por arma de fogo 1 . Pelo mecanismo de trauma, é mais prevalente em homens jovens (mais de 80% são do sexo masculino, com média de idade de 33 anos) 7 ^,^ 8 . Apenas 5% dos aneurismas de ATS não são pseudoaneurismas de origem traumática, sendo classificados como aneurismas ateroscleróticos ou congênitos 9 . 

 A localização mais comum é no ramo anterior da ATS. Raramente está localizado proximalmente ou no ramo posterior. A região do ramo anterior mais comumente lesada é aquela que cruza a proeminência relacionada à junção da fáscia temporal superficial e à linha temporal superior, na porção anterior do crânio. O ramo anterior é literalmente esmagado contra essa proeminência óssea durante o trauma, levando à formação do pseudoaneurisma 8 ^,^ 10 . 

 A apresentação clínica é normalmente benigna, já que a ruptura é rara. Existem apenas três casos de ruptura relatados na literatura 11 ^-^ 13 . A maioria dos pseudoaneurismas (90%) apresenta-se como massa pulsátil assintomática, que pode variar entre 0,5 e 5,7 cm (maioria entre 1 e 1,5 cm de diâmetro). Após o episódio do trauma, o pseudoaneurisma torna-se evidente após duas a seis semanas 1 . Eventualmente, apresenta-se doloroso, causando desconforto local ou uma série de sintomas vagos. Pode estar acompanhado de tontura, alterações visuais ou alterações neurológicas, porém relacionadas ao trauma prévio 1 . Nesses casos, a tomografia computadorizada ou a arteriografia devem ser realizadas para excluir possíveis lesões associadas 13 ^-^ 15 . Na grande maioria das vezes, o exame clínico e a anamnese detalhada dirigida principalmente para a existência de história de trauma recente são suficientes para o diagnóstico. Deve-se realizar sempre a palpação cuidadosa da massa e a oclusão da ATS proximalmente para verificar a diminuição do pulso local, o que ajuda no diagnóstico diferencial. Exames complementares são reservados aos casos em que não existe uma história típica de traumatismo recente 1 ^,^ 8 . 

 Historicamente, vários tratamentos foram utilizados para o tratamento do pseudoaneurisma de ATS, desde a simples compressão 16 até a ligadura proximal da artéria carótida comum 1 . O tratamento deve ser indicado para o alívio dos sintomas locais, por razões estéticas ou para prevenir a ruptura. O tratamento atual é a ressecção do aneurisma após ligadura proximal e distal do ramo anterior sob anestesia local ou geral 5 . O tratamento é seguro e evita a recorrência. A embolização é reservada aos pseudoaneurismas localizados na porção proximal da ATS, onde o acesso cirúrgico é complexo e está acompanhado de risco de lesão da glândula parótida e do nervo facial 17 . O índice de complicação da embolização varia de 1 a 3% e inclui inflamação local acompanhada de dor, trombose induzida pelo cateter, ruptura do aneurisma e embolização acidental da artéria carótida interna 18 . 

 O pseudoaneurisma da ATS, embora raro e de baixa morbidade, deve ser levado em consideração em todo paciente que apresenta história de traumatismo craniano fechado. Todo profissional que atende urgência/emergência deve estar alerta a essa entidade clínica. 
