# Supplementary material for: Pseudoaneurisma da artéria temporal superficial: relato de três casos
Source: J Vasc Bras. 2018 Jan-Mar;17(1):76–80. [Article in Portuguese] doi: 10.1590/1677-5449.009517 (PMC5990264; doi:10.1590/1677-5449.009517)
Supplement: Translation

# Translation: Pseudoaneurysms of the superficial temporal artery: three case reports

**Authors:** Marcio Miyamotto, Matheus Schimidt Evangelista, Victor Hugo Granella, Bruna Zimmerman Angelo, Bárbara Milena Marciniak, Pedro Henrique Hennig, Ian Gimenez Ribeiro, Ricardo César Rocha Moreira

**Affiliations:** 1 Pontifícia Universidade Católica do Paraná – PUC-PR, Hospital Universitário Cajuru – HUC, Serviço de Cirurgia Vascular e Endovascular, Curitiba, PR, Brasil.; 2 Instituto VESSEL de Aperfeiçoamento Endovascular de Curitiba, Curitiba, PR, Brasil.; 3 Hospital Nossa Senhora das Graças – HNSG, Serviço de Cirurgia Vascular e Endovascular Elias Abrão, Curitiba, PR, Brasil.; 4 Pontifícia Universidade Católica do Paraná – PUC-PR, Hospital Universitário Cajuru – HUC, Liga Acadêmica de Medicina Vascular – LAMEV, Curitiba, PR, Brasil.

**Keywords:** temporal arteries, false aneurysm, head traumas

## Abstract

Pseudoaneurysms of the superficial temporal artery are rare events. They primarily occur in young men as a consequence of blunt head traumas. The majority of cases are asymptomatic, although there are sometimes vague symptoms. Ruptured pseudoaneurysms and development of neurological deficits are occasional complications. The authors describe three cases of pseudoaneurysm of the superficial temporal artery after blunt head trauma. Patients were successfully managed with ligature and resection of the pseudoaneurysms.

## INTRODUCTION

 Traumatic pseudoaneurysms of the superficial temporal artery (STA) are rare and account for less than 1% of aneurysms reported. 1 The first case described was reported by Thomas Bartholin in 1740 2 ^,^ 3 and since then approximately 200 cases have been described in the literature. 4 Although associated with low morbidity, a pseudoaneurysm of the STA can cause a series of local symptoms and can even rupture. The authors describe three cases of pseudoaneurysm of the STA secondary to blunt head trauma. 

## DESCRIPTIONS OF THE CASES

### Case 1

 The patient was a 22-year-old male complaining of discomfort in the anterior, right, parietal region associated with an increase in volume at the site. He reported an automobile accident 3 months previously in which he had received a blunt trauma to the right temporal region. He described bleeding at the time of the trauma and subsequent periorbital hematoma. He was assessed and treated with suture of the wound and observation; but after 20 days, he noted a progressive increase in volume at the trauma site. On physical examination, the patient exhibited a pulsating nodule of approximately 1.5 cm in diameter, with no murmur or thrill, and the pulsation reduced in response to proximal manual compression. He was diagnosed with pseudoaneurysm of the STA and arteriography was conducted to investigate the possibility of additional lesions, which confirmed the diagnosis and ruled out intracranial injuries ( Figure 1 a). The patient underwent surgical treatment by ligature and resection of the aneurysm ( Figure 1 b). He recovered well and there was no recurrence of the aneurysm. 

**Figure 1:** Case 1. (a) Arteriography showing pseudoaneurysm of the anterior branch of the superficial temporal artery; (b) Surgical treatment by arterial ligature and resection of the pseudoaneurysm.

### Case 2

 A 27-year-old male patient had suffered direct aggression (a punch) to the anterior parietal area 15 days previously. He developed a painless pulsating mass in the temporal area that was diagnosed as a pseudoaneurysm of the STA and managed with surgical resection and arterial ligature. He recovered well and there was no recurrence of the aneurysm ( Figure 2 ). 

**Figure 2:** Surgical treatment of the second case by arterial ligature and aneurysmectomy.

### Case 3

 This patient was a 29-year-old male who had suffered a high energy automobile accident. While in intensive care because of coma, an increase in volume in the right temporal region was observed and diagnosed as a pseudoaneurysm. He was treated surgically with good results ( Figure 3 ). 

**Figure 3:** Surgical treatment of the third case by arterial ligature and resection of the pseudoaneurysm.

## DISCUSSION

 Since the first description, several series of pseudoaneurysms of the STA have been reported in the literature. Although it was first described as secondary to penetrating trauma, 5 the great majority of cases of STA pseudoaneurysm are the result of blunt traumas. 6 It is associated with participation in several sports, such as hockey, rugby, squash, and baseball. It has also been described in relation to penetrating injuries caused by temporomandibular joint replacement, hair transplants, removal of cysts, lacerations, and gunshot wounds. 1 The trauma mechanism and therefore the condition itself is most prevalent among young men (more than 80% of reports were in males, with a mean age of 33 years). 7 ^,^ 8 Just 5% of aneurysms of the STA are not pseudoaneurysms of traumatic origin and are classified as atherosclerotic or congenital aneurysms. 9

 The most common site is the anterior branch of the STA. They are rarely located proximally or along the posterior branch. The part of the anterior branch that is most often injured is where it crosses the prominence at the junction between the superficial temporal fascia and the superior temporal line, on the anterior part of the skull. The anterior branch is literally crushed against this bony prominence during the trauma, leading to formation of the pseudoaneurysm. 8 ^,^ 10

 Clinical presentation is normally benign, since rupture is rare. There are only three cases of rupture reported in the literature. 11 ^-^ 13 The majority of pseudoaneurysms (90%) present as an asymptomatic pulsating mass, which can range in size from 0.5 to 5.7 cm (the majority are 1 to 1.5 cm in diameter). After the trauma episode, the pseudoaneurysm becomes evident in 2 to 6 weeks. 1 They can occasionally be painful, causing localized discomfort or a series of vague symptoms. There may also be dizziness, visual disturbances, or neurological changes, but these are related to the prior trauma. 1 In these cases, computed tomography or arteriography should be used to rule out possible associated injuries. 13 ^-^ 15 In the great majority of cases, detailed clinical examination and patient history, primarily focused on the possibility of recent trauma, are sufficient to make a diagnosis. Careful palpation of the mass and proximal occlusion of the STA should always be performed to test for a reduction in the pulse, which is useful for differential diagnosis. Additional examinations are only needed if the typical history of recent trauma is absent. 1 ^,^ 8

 Historically, many treatments have been used to mange pseudoaneurysms of the STA, ranging from simple compression 16 to proximal ligature of the common carotid artery. 1 Treatment should be directed at relief of local symptoms, esthetic objectives, or prevention of rupture. Currently, the preferred treatment is resection of the aneurysm after proximal and distal ligature of the anterior branch under local or general anesthesia. 5 This treatment is safe and prevents recurrence. Embolization is reserved for pseudoaneurysms located in the proximal part of the STA, where surgical access is complex and there is risk of injury to the parotid gland and facial nerve. 17 The complication rate of embolization ranges from 1 to 3% and conditions include local inflammation with pain, thrombosis caused by the catheter, rupture of the aneurysm, and accidental embolization of the internal carotid artery. 18

 Although a pseudoaneurysm of the STA is rare and has a low rate of morbidity, it should be considered in all patients with a history of blunt head trauma. All professionals who work in emergency should be aware of this clinical entity.
